# Data survey on the antecedent of the entrepreneurial intention in Indonesia

**DOI:** 10.1016/j.dib.2019.104317

**Published:** 2019-07-25

**Authors:** Tony Wijaya, Sukidjo, Sunarta

**Affiliations:** Universitas Negeri Yogyakarta, Indonesia

**Keywords:** Risk-taking propensity, Self-efficacy, Entrepreneurial attitude, Entrepreneurship intentions

## Abstract

This article presents data that examine the effect of the self-efficacy on the risk-taking propensity, risk-taking propensity and self-efficacy influences affect the entrepreneurial attitude, and the effect of entrepreneurial attitude toward the entrepreneurship intentions. Data collected using a questionnaire adapted from previous scholars. The sample in this research was prospective entrepreneurs who take part in Indonesia entrepreneurship programs and 315 respondents were collected. Simple random sampling technique was applied, validity and reliability procedures were also confirmed. The structural equation modeling was used.

**Table:** Specifications Table

Subject area	*Business, Management*
More specific subject area	*Business, Entrepreneurship.*
Type of data	*Table, figures*
How data was acquired	*Survey with questionnaire*
Data format	*Raw, analyzed statistical data*
Experimental factors	*Samples consist of prospective entrepreneurs who take part in Indonesia entrepreneurship programs*
Experimental features	*Data were analyzed using structural equation modeling*
Data source location	*Java, Indonesia.*
Data accessibility	http://staffnew.uny.ac.id/upload/197907162014041001/lainlain/Raw&output.pdf
Related research article	S. Gaddam, Identifying the relationship between behavioral motives and entrepreneurial intentions: an empirical study based participations of business management students, *The Icfaian Journal of Management Research*., 7, 2008, 35–55 [1].

**Table:** 

**Value of the data** • This data present useful information on the antecedent of the entrepreneurial intention i.e. the effect of the self-efficacy on the risk-taking propensity, risk-taking propensity, and self-efficacy influences affect the entrepreneurial attitude and the effect of entrepreneurial attitude toward the entrepreneurship intentions. This data reflects the entrepreneurial intention in Indonesia. This survey can be taken into consideration in the replication of research in other countries. • This data also presents information on the importance of entrepreneurship programs based on self-efficacy and risk-taking propensity. Stakeholders who want to develop entrepreneurship training programs can consider antecedent aspects to realize entrepreneurial intentions. The government can consider this data analysis as useful information in encouraging the number of an entrepreneur through entrepreneurial intention antecedents. • This data is useful for researchers who are interested in developing entrepreneurship from behavioral or psychological aspects. The data analysis can be used as a comparison with other studies with a similar perspective.

## 1 Data

**Fig. 1:**
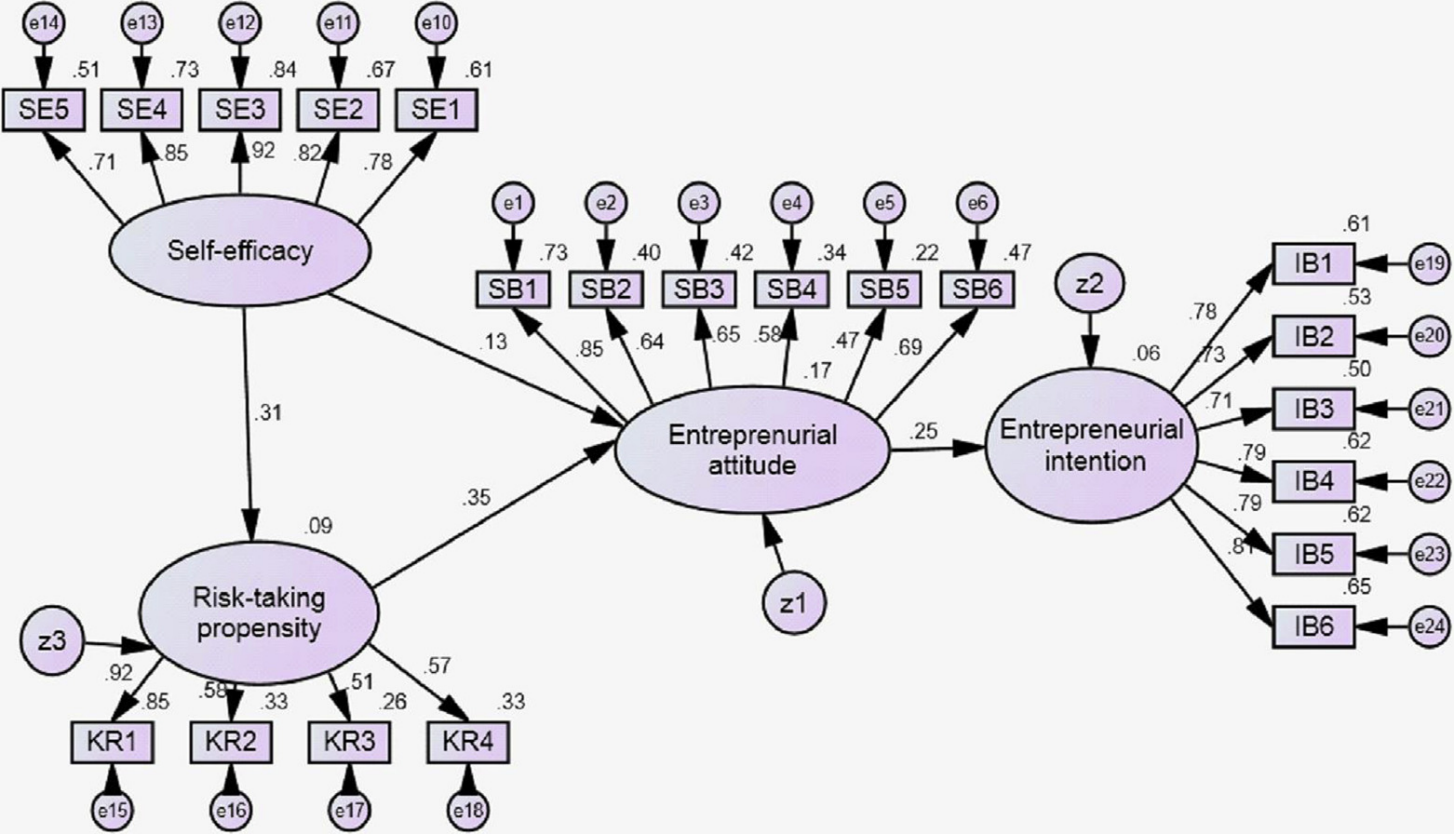
Path model.

The data provided the information of the antecedent of the entrepreneurial intention i.e. the effect of the self-efficacy on the risk-taking propensity, risk-taking propensity and self-efficacy influences affect the entrepreneurial attitude and the effect of entrepreneurial attitude toward the entrepreneurship intentions. The method of data analysis in this study used structural equation modeling. Data that has been collected is tested for normality first. Data is said to be normal if the multivariate c.r (critical ratio) has a requirement of −2.58 < c.r < 2.58 [6]. The results of the normality test show normal data with multivariate c.r of 2.19 < 2.58 so that all data can be processed further. The collected data was tested for validity and reliability. Overall the value of the load factor (factor loading) of each variable is observed so that it can be concluded that all observed variables of the latent variables are valid and meet the criteria of the measurement model that is methodologically fit. Reliability diagnostic measure is the reliability coefficient. The generally agreed upon lower limit for Cronbach's alpha is 0.70 [6]. Table 1 shows the value of the factor load measured from latent variables through each observed variable and reliability coefficient. The fit of model test results using chi-square, CMIN/DF, GFI, AGFI, RMSEA, TLI, and CFI are summarized in Table 2. All model fit indices match with recommended literature cut-off values [6]. The results of the causality test between variables in the model are shown in Table 3 and Fig. 1. With a 5% probability acceptance limit, the results show that self-efficacy influences the risk-taking propensity, the risk-taking propensity influences entrepreneurial attitudes, but self-efficacy does not significantly influence entrepreneurship attitudes, and entrepreneurial attitudes influence entrepreneurial intentions. The results of this finding are supported by previous research [1], [2], [3], [4]. Table 4 showing a value of direct and indirect effects, and the total effect.

**Table 1:** Factor loading and reliability.

Variable	λ_i_	Reliability
Risk-taking propensity:		0,73
KR1	0,92	
KR2	0,58	
KR3	0,51	
KR4	0,57	
Entrepreneurial attitude:		0,81
SB1	0,85	
SB2	0,64	
SB3	0,65	
SB4	0,58	
SB5	0,47	
SB6	0,69	
Self-efficacy:		0,91
SE1	0,78	
SE2	0,82	
SE3	0,92	
SE4	0,85	
SE5	0,71	
Entrepreneurship intentions:		0,89
IB1	0,78	
IB2	0,73	
IB3	0,71	
IB4	0,79	
IB5	0,79	
IB6	0,81

**Table 2:** The goodness of fit statistics.

Index	Cut off Value	Result	Model evaluation
Chi-square	Approach 0	273,780	Good
Probability	≥0,05	0,058	Good
CMIN/DF	≤2,00	1,210	Good
GFI	≥0,90	0,942	Good
RMSEA	≤0,08	0,044	Good
AGFI	≥0,90	0,906	Good
TLI	≥0,90	0,957	Good
CFI	≥0,90	0,962	Good

**Table 3:** Regression weights.

Variable			Estimate	S.E.	C.R.	P
Risk-taking propensity	←	Self-efficacy	0,170	0,066	2,597	0,009
Entrepreneurial attitude	←	Risk-taking propensity	0,447	0,156	2,859	0,004
Entrepreneurial attitude	←	Self-efficacy	0,094	0,077	1,224	0,221
Entrepreneurship intentions	←	Entrepreneurial attitude	0,316	0,141	2,247	0,025

**Table 4:** Direct, indirect and total effect.

Independent Variable	Dependent Variable	Direct effect	Indirect effect	Total effect
Entrepreneurial attitude	Entrepreneurship intentions	0,247	–	0,247
Risk-taking propensity	Entrepreneurship intentions	–	0,087	0,087
Self-efficacy	Entrepreneurship intentions	–	0,060	0,060
Risk-taking propensity	Entrepreneurial attitude	0,351	–	0,351
Self-efficacy	Entrepreneurial attitude	0,133	0,108	0,241
Self-efficacy	Risk-taking propensity	0,307	–	0,307

## 2 Experimental design, materials, and methods

The data was based on quantitative analysis. The method of analyzing data in this study used structural equation modeling. This statistical tool can be used to test the fitness of the model and the relationship between variables that are multivariate both directly and indirectly. Structural equation modeling provides the appropriate and most efficient estimation of the technique for a series of separate multiple regression estimated simultaneous equations [6].

The samples collected were 315 respondents, namely prospective entrepreneurs who take part in Indonesia entrepreneurship programs. The criteria for respondents are prospective entrepreneurs who have participated in entrepreneurship training programs and already have plans for entrepreneurship. The data collecting instrument that has already been developed and adapted from previous scholars [1], [2], [3], [4], [5]. Questions had a 5-point Likert scale from Strongly disagree (1) to Strongly agree (5) in an orderly manner that measures respondents' attitudes by measuring the extent to which they agree or disagree with certain questions or statements. Data is collected with permission from respondents and has been explained previously about the purpose of the research and the use of this research data. Respondents were asked to participate in data collection and were given a brief explanation that the data obtained would be used for scientific and academic development purposes. The data was collected protecting confidentiality and anonymity of the respondents. The ethical code was obtained from the economic faculty of Yogyakarta State University. Data is processed using Amos-20.
